# Future Impact of mRNA Therapy on Cardiovascular Diseases

**DOI:** 10.14797/mdcvj.1169

**Published:** 2022-12-06

**Authors:** John P. Cooke, Keith A. Youker

**Affiliations:** 1 Houston Methodist Research Institute, Houston, Texas, US

**Keywords:** myocardial infarction, heart failure, arrhythmias, hypercholesterolemia, senescence, telomerase, vaccine

## Abstract

The silver lining of the recent pandemic was that it accelerated the emergence of messenger ribonucleic acid (mRNA) therapeutics. The great promise of mRNA therapeutics was highlighted by the speed at which the vaccines were created, tested, and proven to be relatively safe and highly effective. There are a wide variety of mRNA therapeutics now under development, and dozens of these are in clinical trials. These therapeutics are generating a major paradigm shift in medical therapy, including the treatment of cardiovascular disease. Most of the cardiovascular mRNA therapies are still in preclinical development, although a phase 2a trial of mRNA therapy for myocardial ischemia has been completed with promising results.^1^ The application of mRNA therapies to cardiovascular diseases is virtually limitless, and ongoing work includes mRNA therapies for myocardial ischemia, heart failure, arrhythmias, hypercholesterolemia, and arterial occlusive diseases. In addition, mRNA may be used to enhance cell therapies. In the future, mRNA therapies for cardiovascular disease are destined to supplant some of our current biologics and pharmacotherapies and will be used to treat previously untreatable cardiovascular diseases. Furthermore, mRNA therapies can be personalized, and they can be rapidly generated in current Good Manufacturing Practice facilities with a modest footprint, facilitating the rise of hospital-based regional centers of RNA therapeutics.

## The RNA World

There is accumulating evidence for the notion that life began in an RNA world. This hypothesis states that RNA (ribonucleic acid) existed long before proteins and DNA (deoxyribonucleic acid) emerged on planet Earth. The chemistry of primitive Earth would have supported the generation of ribonucleotides, followed by their linkage into strands of RNA that provided the structure and function for life to form.^2^ Like DNA, RNA can store information. Unlike DNA, which tends to form highly stable double helices, RNA is more labile, reactive, and forms a greater variety of structures. Although RNA is typically a single-stranded biopolymer, self-complementary sequences in the RNA molecules facilitate intrachain base pairing and folding of the chain into double or triple helices, bulges, cloverleafs, and a variety of other structures. Such structural variety provides disparate RNA molecules with catalytic properties needed for life (ribozymes), the ability to sense and react to changes in the environment (riboswitches), the capacity to bind to other RNA molecules and organize complex structures (such as seen with long non-coding RNA), and the ability to self-replicate (RNA polymerase ribozymes).^3^

The early RNA lifeforms may explain the current diversity of RNA molecules that regulate contemporary life. Messenger RNA (mRNA) is a transient copy of a portion of the DNA template that encodes a protein necessary for cell function or structure. The translation of mRNA into protein requires transfer RNA (tRNA) and ribosomal RNA (rRNA), which respectively carry amino acids to the ribosomal machinery and catalyze their linkage into a peptide chain. A great diversity of small RNAs (small nuclear RNA, small nucleolar RNA, microRNA, small interfering RNA) act on the pre-mRNA molecule to facilitate its splicing into the mature mRNA molecule, the transport of mature mRNA throughout the cell, and the silencing or degradation of mRNA. Other RNA molecules regulate chromosomal DNA. For example, piwi-interacting RNA (piRNA) silence transposons (jumping genes in the chromatin, originally derived from viral elements) and defend against viral infections.^4^ Long noncoding RNA (lncRNA) can form scaffolds to connect transcriptional factors with a promoter sequence on chromosomal DNA to initiate transcription.^5^ Each of these many forms of endogenous RNA are potential targets for therapeutic modulation.

## The First RNA Drugs for Cardiovascular Disease

This review focuses on the promise of mRNA therapeutics for cardiovascular diseases. However, the first RNA drugs to reach the market were small interfering RNAs that reduce the expression of a particular mRNA target and are worthy of brief mention in this review. Among these interference RNA (RNAi), the first to reach clinical approval for treatment of a cardiovascular disease was an RNAi against transthyretin known as patisiran (Onpattro). The RNAi are silencing RNA that bind to the 3’ untranslated portion of the mRNA to block its translation and/or to accelerate its degradation. In this case, patisiran accelerated the degradation of normal or mutated transthyretin (TTR) that caused TTR amyloidosis. TTR amyloidosis is a multisystemic life-threatening disease caused by a mutation in the gene encoding TTR, causing deposits of amyloid in peripheral nerves, the heart, kidney, and other organs.^6^ In a phase 3 trial using RNAi to inhibit mRNA expression of a mutated TTR gene, multiple clinical manifestations were improved.^7^ The APOLLO phase 3 trial showed an increase in the quality of life of patients with TTR amyloidosis.^8^ Patisiran reduced left ventricular wall thickness and longitudinal strain as well as levels of brain natriuretic peptide (a marker of impaired cardiac function). In 2018, patisiran became the first siRNA approved by the United States (US) Food and Drug Administration (FDA) and has opened the door for treating other diseases related to a single protein mutation.

The second interference RNA to gain FDA approval (in December 2021) was the siRNA inclisiran, which targets mRNA encoding proprotein convertase subtilisin/kexin type 9 serine protease (PCSK9). This molecule downregulates the receptor for low-density lipoprotein (LDL-R). Inclisiran reduces serum levels of LDL cholesterol because it silences the mRNA encoding PCSK9 and thereby reduces PCSK9 expression.^9^ The reduced expression of PCSK9 causes a reciprocal increase in LDL-R and a concomitant reduction on serum LDL cholesterol levels. Inclisiran met all primary and secondary end points across three phase 3 trials, had a favorable safety profile, and matched the LDL-lowering efficiency of antibody-based PCSK9 inhibitors alirocumab (Praluent) and evolocumab (Repatha) after a twice-annual subcutaneous injection.^10,11^ Additional RNAi drugs for the treatment of hypercholesterolemia are currently under development.

## mRNA Drugs: A Revolutionary Paradigm in Therapeutics

**Figure 1:**
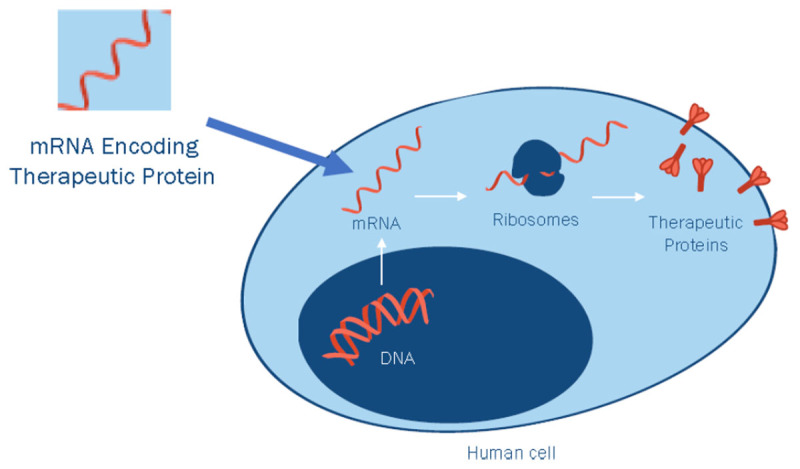
Transcription of endogenous mRNA, or transfection with exogenous mRNA, and its translation into proteins. Chromosomal DNA in the nucleus is transcribed into native mRNA by RNA polymerase. The mRNA that is generated is spliced and processed into the mature mRNA form that is transported into the cytoplasm. There it is translated into protein by the ribosomal machinery. Exogenous mRNA encoding a therapeutic protein crosses the cytoplasm with the aid of transfection procedures such as lipid nanoparticles or electroporation. In the cytoplasm, the ribosomal machinery translates the exogenous mRNA into the therapeutic protein.

Essentially, mRNA is biological software that encodes all the functional and structural proteins of the organism. The mRNA is generated in the nucleus of the cell by a transcription complex that copies a portion of the DNA code into mRNA (Figure 1). The mRNA is processed in the nucleus into a mature form that is transported into specific sites of the cytoplasm. At those sites, the mRNA is translated into protein by the ribosomal machinery. Each mRNA can be used to make tens to hundreds of molecules of the protein encoded by that mRNA. However, mRNA is degraded by nucleases within minutes to hours. The mRNA is meant to be a transient message, instructing the cell to make the protein that is needed at that specific time in a specific region of the cell. The transient nature of the message provides the cell with a flexible response system, permitting the protein repertoire of the cell to change rapidly in response to a challenge.

If one can introduce an exogenous mRNA molecule into the cell, the mRNA will be acted upon by the ribosomal machinery to generate the protein encoded by that exogenous mRNA molecule. As discussed below, methods have been developed to introduce mRNA into cells (eg, lipid nanoparticles). Accordingly, if one wishes to have the patient’s cells produce a particular therapeutic protein, one can essentially write the code for that protein into the mRNA molecule and deliver the mRNA molecule to the patient’s cells (Figure 1).^12^

Because mRNA is biological software, one can rapidly write the code for a therapeutic protein. The speed of mRNA drug development was dramatically highlighted by the COVID-19 pandemic. Traditionally, it has taken about a decade or more to gain approval for a novel vaccine. By stark contrast, the mRNA vaccine against SARS-CoV-2 (Severe Acute Respiratory Syndrome-associated coronavirus 2) attained emergency use authorization in less than a year.^13^ In January 2020, Chinese scientists published the SARS-CoV-2 genome. Within days of knowing that biological code, biotech and pharmaceutical companies Moderna and BioNTech/Pfizer were able to design and synthesize mRNA constructs encoding the spike protein of the virus. These mRNA constructs were tested rapidly in preclinical models, and within months the first human trials began. There is a current global effort to further accelerate our response to the next pandemic, in part by using an RNA platform.^14^

To be sure, previous scientific work with other lethal coronaviruses (eg, the prior SARS-CoV as well as the coronavirus causing Middle Eastern Respiratory Syndrome, or MERS) provided critical information that accelerated the development of the vaccine against SARS-CoV-2.^15^ From earlier work it was known that neutralizing antibodies against the spike protein of these coronaviruses played an essential role in immune protection, and that recombinant spike protein or DNA encoding spike protein could generate a protective immune response against the coronaviruses.^16^ Furthermore, it became clear that it was necessary to mutate two of the amino acids in the spike protein to maintain the protein in its prefusion conformation so that immune recognition of the antigen was enhanced. This earlier work accelerated the development of an mRNA coronavirus vaccine. In addition, there were some general advances in the understanding of mRNA biology, and its delivery, that have made mRNA therapeutics possible.

## The Emergence of mRNA Therapeutics

One might ask why mRNA therapies are making their appearance only now, 60 years after the discovery of mRNA. To be sure, preclinical studies of mRNA therapies date back to the 1990s, when it was shown that mRNA encoding vasopressin, injected into the hypothalamus of Brattleboro rodents, could generate vasopressin and transiently reverse diabetes insipidus in these animals. However, synthetic mRNA also triggered a strong inflammatory response and caused cytotoxicity. It was not until 2005, when Drew Weissman and Katalin Kariko published their landmark paper, that it became generally recognized that base modifications in mRNA were essential to avoid activation of pattern recognition receptors (PRRs) in mammalian cells.^17^ These PRRs recognize viral RNA and trigger inflammatory signaling that leads to the release of inflammatory cytokines, suspends translation of protein in the cells, and can even induce cell death. Weissman and Kariko found that human RNA contained base modifications that permitted escape from recognition by PRRs. When they synthesized mRNA using modified bases (eg, replacing uracil with pseudouridine) the excessive inflammatory signaling of mammalian cells that was generated in response to exogenous mRNA was markedly reduced. As a result, cell survival was improved, and the translation of the mRNA into protein was enhanced. In addition, these investigators showed that better purification strategies (to remove abortive transcripts and double-stranded RNA) also attenuated excessive inflammatory signaling and cytotoxicity.

Improvements in delivery of mRNA also contributed to the emergence of mRNA therapeutics. Naked mRNA is very fragile, subject to hydrolytic degradation in aqueous solution and rapidly degraded by exonucleases. The transient nature of mRNA permits a cell to rapidly modify its transcriptional profile in response to changes in its environment. However, the ephemeral nature of mRNA makes it a difficult therapeutic to deliver. The development of lipid nanoparticles made mRNA therapies more feasible.^18^ By protecting the mRNA in a lipid nanoparticle, it is protected from hydrolytic degradation as well as enzymatic degradation by exonucleases that are ubiquitous in tissue. Furthermore, the lipid nanoparticle facilitates passage of the mRNA through the cell membrane via endocytosis.

## Novel Cardiovascular mRNA Therapies in Development

### Reversing Myocardial Ischemia

Vascular endothelial growth factor (VEGF) is a potent facilitator of angiogenesis. In preclinical models, administration of VEGF as a recombinant protein or a gene therapy has increased microvascular density, improved perfusion to ischemic tissue, and improved function. However, clinical trials of VEGF as recombinant protein or gene therapy for myocardial or limb ischemia have failed. For example, VEGF-A delivery by plasmid (NOTHERN trial^19^) or adenovirus (KAT trial^20^) using intramyocardial delivery provided no significant clinical benefit. Nevertheless, based upon promising data in a murine model of myocardial ischemia—where injections of naked VEGF mRNA could increase local levels of VEGF, the infiltration of progenitor cells, and microvascular density and could reduce myocardial damage^21^—VEGF mRNA for myocardial ischemia has undergone clinical development. A phase 1 study in human volunteers with diabetes mellitus was promising.^22^ In this unique study design, VEGF mRNA was injected subcutaneously, and VEGF protein was detected by cutaneous microdialysis. Injections of VEGF mRNA in a sodium citrate buffer induced dose-dependent increases in VEGF protein expression. Furthermore, laser Doppler perfusion studies revealed an increase in local perfusion. These promising studies led to a phase 2a trial in patients undergoing coronary artery bypass grafting (CABG). At the time of the CABG, surgeons delivered mRNA by intramyocardial injection at 30 sites in the ischemic region. Follow-up studies showed a reduction in plasma levels of brain natriuretic peptide and an increase in walking distance on a treadmill.^23^ Whereas these studies show promising proof of concept, it is likely that this therapeutic approach will be improved by a better delivery vehicle and/or more stable RNA design.

### Restoring Myocardial Functions

In addition to improving cardiac perfusion, increasing myocyte regeneration is necessary for recovery from myocardial injury. The transcriptional factor Yes-associated protein (YAP) binds to the promoters of a cohort of genes that are necessary to reactivate myocyte dedifferentiation and proliferation. It is the activated form of YAP (aYAP) that translocates from the cytoplasm to the nucleus to mRNA to induce myocardial regeneration. In a murine model of myocardial infarction, it has been shown that intramyocardial injections of aYAP mRNA can increase the local expression of this transcriptional factor, spark myocyte proliferation, increase viable myocardium and reduce scar, and improve cardiac function.^24^

In addition to restoring cardiac contractile function, it is necessary to maintain normal electrophysiological function. In patients with sick sinus syndrome (SSS) and other disorders of the sinoatrial (SA) or atrioventricular (AV) nodes, the loss of endogenous pacemaker activity often requires the placement of a pacemaker device. Whereas permanent pacemaker devices are extraordinarily effective, they can fail in many ways, such as failing to sense the endogenous rhythm, stimulate the myocardium, and/or induce dysrhythmias. Fragmentation of the pacemaker leads, generator or battery failure, and infection are recognized problems of permanent pacemakers. The vision of a biological pacemaker that would avoid such problems, and would replace a dysfunctional SA or AV node, has led to cell and gene therapeutic approaches to reconstitute a normally functioning endogenous pacemaker. Recent advances in cellular reprogramming have led to the recognition that there are a few master regulators of cell fate. The expression of specific transcriptional factors, together with an increase in DNA accessibility, can change cell identity. The master regulator of pacemaker cell identity in the AV node is TBX18. When overexpressed in a cardiac myocyte, TBX18 can induce the genes required for a myocyte to have the electrophysiological properties of an AV nodal cell. Recently, mRNA encoding TBX18 has been shown to induce pacemaker function in cardiac myocytes and restore normal rhythm in a rodent model of AV nodal block.^25^ As with the other mRNA therapies for cardiovascular disease mentioned above, more stable forms of mRNA and better delivery vehicles will make these drugs feasible for clinical trials.

### Turning Back the Clock on Cardiovascular Senescence

The telomeres at the ends of each chromosome consist of thousands of hexanucleotide repeats and play a major role in chromosomal integrity. The telomere folds back on itself and is shrouded by shelterin proteins, which prevent the DNA damage machinery from recognizing the ends of the chromosomes as breaks in need of repair. With each cell division, the telomeres become shorter due to the “end-replication problem.” In addition, reactive oxygen species can induce telomeric damage that leads to accelerated shortening. Disease states that are associated with increased inflammation and oxidative stress, such as diabetes mellitus, are also associated with telomere shortening.

In embryonic stem cells, the expression of telomerase protein maintains telomere length, permitting indefinite cell division. To a lesser extent in adult stem cells (such as mesenchymal stem cells, or MSCs), telomerase is expressed, permitting greater replicative capacity of these stem cells than normal somatic cells. In somatic cells, telomerase is generally not expressed. For this reason, differentiated cells (such as skin, cardiac, or vascular cells) have a limited lifespan. For example, human fibroblasts have the capacity to double about 50 times, then they become senescent and incapable of further proliferation, a barrier that is known as the Hayflick Limit. Importantly, senescent cells also produce inflammatory cytokines that make the other cells around them dysfunctional.

We have shown that mRNA encoding telomerase (TERT) can transiently increase cellular expression of telomerase protein.^26^ Although the mRNA is only present for a few hours and the telomerase protein is only detectable for 48 to 72 hours, we observe appreciable increases in the length of the telomere. This is a persisting structural change in the chromosomes of the cells that lasts much longer than does the RNA or protein telomerase. This structural alteration in the telomeres is associated with an increase in replicative capacity. We have shown that four treatments with mRNA TERT can double the lifespan of a human fibroblast,^27^ and that this increase in replicative capacity also occurs for endothelial cells, myoblasts, and keratinocytes.

In addition to an increase in replicative capacity, we observe a stunning reversal of many of the abnormalities observed in senescent cells. For example, endothelial cells derived from patients with progeria are very abnormal. Like the children with this disease, the endothelial cells grow poorly and die early. They have an abnormal morphology, the classic “fried egg” appearance of senescent endothelial cells, with abnormally lobulated nuclei. The progeria endothelial cells are dysfunctional, with impaired generation of nitric oxide, reduced capacity to form vascular networks in Matrigel, and reduced uptake of acetylated LDL cholesterol. They generate inflammatory cytokines and express adhesion molecules (eg, vascular cell adhesion molecule) typical of aberrantly activated endothelial cells. The transcriptional profile is consistent with senescence, the telomeres are shorter, and there is evidence of DNA damage. This global alteration of endothelial cells from the progeria patients explains in part why they succumb to heart attack and stroke in their mid-teens.

Remarkably, two treatments with mRNA TERT reverses or ameliorates these alterations. Together with extension of the telomeres, there is a reduction in DNA damage signals, a normalization of the nuclear morphology and the transcriptional profile, and an improvement in cellular functions and replicative capacity. In a murine model of progeria, mice treated with a lentiviral vector encoding telomerase had less vascular DNA damage and inflammatory markers and lived longer, providing further rationale for a strategy of reversing senescence using mRNA TERT.^28^ However, the development of mRNA TERT for reversing cardiovascular senescence (and other age-related diseases) will require improved delivery vehicles.

### Enhancing Cell Therapies

Cell therapy for cardiovascular disease, while appearing promising in preclinical studies, has disappointed in clinical trials. One of the problems with autologous cell therapies (eg, adipose or bone marrow-derived MSCs) is that the stem cells derived from the diseased patient may themselves be diseased. If one understands the mechanisms by which the stem cells are deficient, one can design an mRNA construct to transiently modify the stem cell so that it can better perform its regenerative function. In this regard, an mRNA construct could encode survival factors that would permit more efficient tissue integration, or growth factors that would provide for greater regenerative efficacy. Another approach would be to rejuvenate the stem cells from the diseased patient using mRNA encoding TERT.

Whereas we have not yet used mRNA TERT to improve cardiovascular cell therapy, we have used it to improve a regenerative product for burn patients. Avita Medical has a cell therapy product for burn patients. Rather than meshing the donor skin for direct application to the burn site, the Avita device disaggregates the human skin into single cells that are then sprayed onto the burn site. In younger individuals, the cosmetic result is superior to that of standard skin grafting. However, in older individuals, the approach could be improved. Accordingly, we have tested the addition of TERT mRNA in lipid nanoparticles (LNPs) to the disaggregated human skin cells prior to their application. In a wound model in immunodeficient mice (which do not reject the human skin), our preliminary studies indicate that TERT mRNA LNPs added to the disaggregated human skin cells increase their telomerase activity (unpublished results). This effect is associated with an increase in the number of human skin cells in the wound 1 week after application, as well as a reduction in senescence markers and DNA damage signals, and an increase in proliferation markers. Thus we have early proof of concept that TERT mRNA LNPs can enhance cell therapy.

### Vaccines Against Heart Failure

Chagas disease is the major cause of heart failure in South America. The disease is spread by an insect vector, known colloquially as the “Kissing Bug” (Triatominae) or the more apt “Vampire Bug” that bites people around the mouth or eyes during sleep. To add insult to injury, they defecate near the wound, which inoculates the pathogen *Trypanosoma cruzi* (*T. cruzi*). The parasite invades the local tissue, replicates, and bursts out of those cells and into the blood stream, where it infects other organs including the heart. With Dr. Peter Hotez at Baylor College of Medicine, we have generated a bivalent vaccine against *T. cruzi*, and preliminary data seem promising in the Baylor murine model of Chagas disease.^29^ Clinical development of this vaccine seems warranted, particularly since the vector is spreading north into the southern United States.

Another novel vaccine for heart failure has originated from the observation that the chaperonin protein, HSP60 (heat shock protein 60) is aberrantly expressed in heart failure. This chaperonin protein is normally expressed in the cytoplasm and assists in normal protein folding and transport. However, in heart failure, HSP60 translocates to the cell membrane and induces inflammatory signaling and apoptosis.^30^ We have observed increased expression of HSP60 in failing human hearts and detectable levels of HSP60 circulating in the blood. These observations led us to hypothesize that aberrant and increased expression of HSP60 might contribute to heart failure. In our murine model of heart failure (induced by chronic infusion of angiotensin II and the nitric oxide synthase antagonist L-NAME, together with a high salt diet), we observed increased expression of HSP60 in the failing heart and could detect circulating HSP60. A recombinant protein vaccine against HSP60 prevented heart failure in this mouse model.^31^ Animals given the vaccine had substantially improved cardiac function and chamber size. On the basis of these encouraging findings, we are performing additional clinical studies on the association of circulating HSP60 in human heart failure as well as preclinical testing of an mRNA vaccine against circulating HSP60.

### Familial Hypercholesterolemia

Whereas inclisiran can reduce the expression of mRNA encoding PCSK9 and thereby increase LDL-R expression and LDL-cholesterol clearance, another approach would be to deliver a therapeutic neutralizing antibody against PCSK9. Indeed, this is the rationale underlying the LDL cholesterol-lowering therapies alirocumab (Praluent) and evolocumab (Repatha). However, these are recombinant proteins. We have generated mRNA encoding a neutralizing antibody against PCSK9 and have documented the in vitro secretion of the antibody by HepG2 cells. Furthermore, preliminary data indicates that the generation of these mRNA-encoded antibodies abrogates the effect of exogenous PCSK9 protein to reduce hepatic LDL-C uptake.

Patients with homozygous familial hypercholesterolemia have no functional LDL receptors and consequently have exceedingly high levels of LDL cholesterol and succumb to heart attack or stroke as young adults. For these individuals, therapies targeting only PCSK9 are not helpful. Accordingly, we have generated mRNA constructs encoding LDL receptors, including those that lack the PCSK9 binding site. Once expressed in hepatic cells, the mutant LDL receptors encoded by mRNA are resistant to the action of PCSK9 and have prolonged expression. Their expression is associated with increased LDL uptake by the hepatic cells. Further studies are required to determine the feasibility of this approach to treating familial hypercholesterolemia.

## The Path Ahead for Cardiovascular mRNA Therapeutics

A number of exciting new developments will increase the feasibility of broader applications of mRNA therapeutics.

### Augmenting Effect

Self-amplifying RNAs (saRNAs) can replicate themselves. Such RNA molecules can deliver the message to generate a target protein and can encode an RNA-dependent polymerase that copies the RNA molecule. Compared with standard mRNA, less RNA is delivered to a tissue to produce the same amount of therapeutic protein.^32^ The saRNA constructs are based upon the alphavirus, a positive-sense single-stranded RNA virus that can self-replicate well. The standard saRNA thus has two functional domains, one encoding the therapeutic protein and one encoding the RNA-dependent RNA polymerase-encoding sequence along with other elements required for replication. One of the major developmental hurdles for saRNA constructs is their large size (about 10 kB). In vitro synthesis of such large constructs is bedeviled by abortive transcripts as well as fragmentation of the large saRNA during processing. A trans-amplifying RNA partly addresses these issues by separating the functions using two RNA constructs. One construct encodes the desired protein and the other encodes the self-amplification machinery, with both constructs delivered together to achieve self-amplification.^33^

### Increasing Stability

One of the characteristics of mRNA is its lack of stability, lasting for minutes to hours before it is degraded. For some applications, this is useful. For example, the transient nature of the mRNA telomerase construct increases safety. The mRNA lasts a few hours, and the telomerase protein that is translated from the mRNA lasts 48 to 72 hours, which is sufficient to induce an appreciable increase in the telomere that persists long after the telomerase protein has disappeared. Thus the mRNA construct has a sustained benefit without the risk of immortalization. Similarly, the mRNA vaccine construct transiently generates an antigen, but the brief presentation of the antigen to the immune system is sufficient to trigger the generation of neutralizing antibodies, T cell cytotoxicity, and T memory cells that provide immune protection.

For many other applications, however, the ephemeral nature of mRNA is problematic. One can modestly increase the stability of the mRNA by codon optimization, by tissue-specific selection of 3’ and 5’ untranslated regions, and by using a long polyadenylated tail. For example, one can design “smart RNA” based on the library of miRNA of a specific tissue. In this case, one incorporates a 3’ untranslated region sequence that facilitates miRNA silencing in most tissues except the one in which expression is desired.

Structural modifications to mRNA are more likely to substantially increase lifespan of the construct. For example, circularization of the mRNA markedly increases stability. The circular RNA is resistant to 3’ and 5’ exonucleases because it doesn’t have an end. An internal ribosome entry site permits the ribosomal machinery to latch on to the circular RNA and translate it into protein. Circular RNA can persist for days and perhaps weeks. Such constructs can generate therapeutic proteins for a longer period, which will be necessary for treating chronic diseases. A recent study has used circular RNA encoding an insulin receptor to significantly protect the heart from chronic doxorubicin-mediated cardiotoxicity in a mouse model.^34^

### Enhancing Delivery

The currently available lipid nanoparticles (LNPs) work very well for vaccines. Indeed, there is some evidence that the LNPs may act as an adjuvant by stimulating local inflammatory signaling. Whereas this low level of inflammatory signaling can enhance the action of a vaccine, it is not desirable for treating chronic disease. Furthermore, LNPs are largely sequestered in the liver and spleen when delivered systemically. This feature may be useful in treating hepatic disease but is not desirable for treating other organs.

Accordingly, substantial effort is now being directed toward improving LNPs by reducing toxicity and improving tissue distribution. In this regard, we have developed leukosomes, which are LNPs with membrane proteins of autologous leukocytes embedded within the outer membrane of the LNP. Biodistribution studies indicate that leukosomes are preferentially incorporated at sites of inflammation. We have shown that the leukosomes are superior to standard LNPs in delivering therapeutic cargo to atheromatous lesions in the apo E deficient hypercholesterolemic mouse.^35^ Another approach is to label the external surface of the LNP with a targeting antibody. An example of this method was strikingly demonstrated by the Epstein group, who labeled LNPs with an antibody directed against T cells to deliver mRNA encoding a chimeric antigen receptor against activated fibroblasts.^36^ In a preclinical model of cardiac fibrosis, this LNP cargo generated CAR-T cells that diminished the number of activated fibroblasts in the heart, reduced cardiac fibrosis, and improved cardiac function.

## Another Paradigm Shift: Hospital-Based RNA Therapeutics

**Figure 2:**
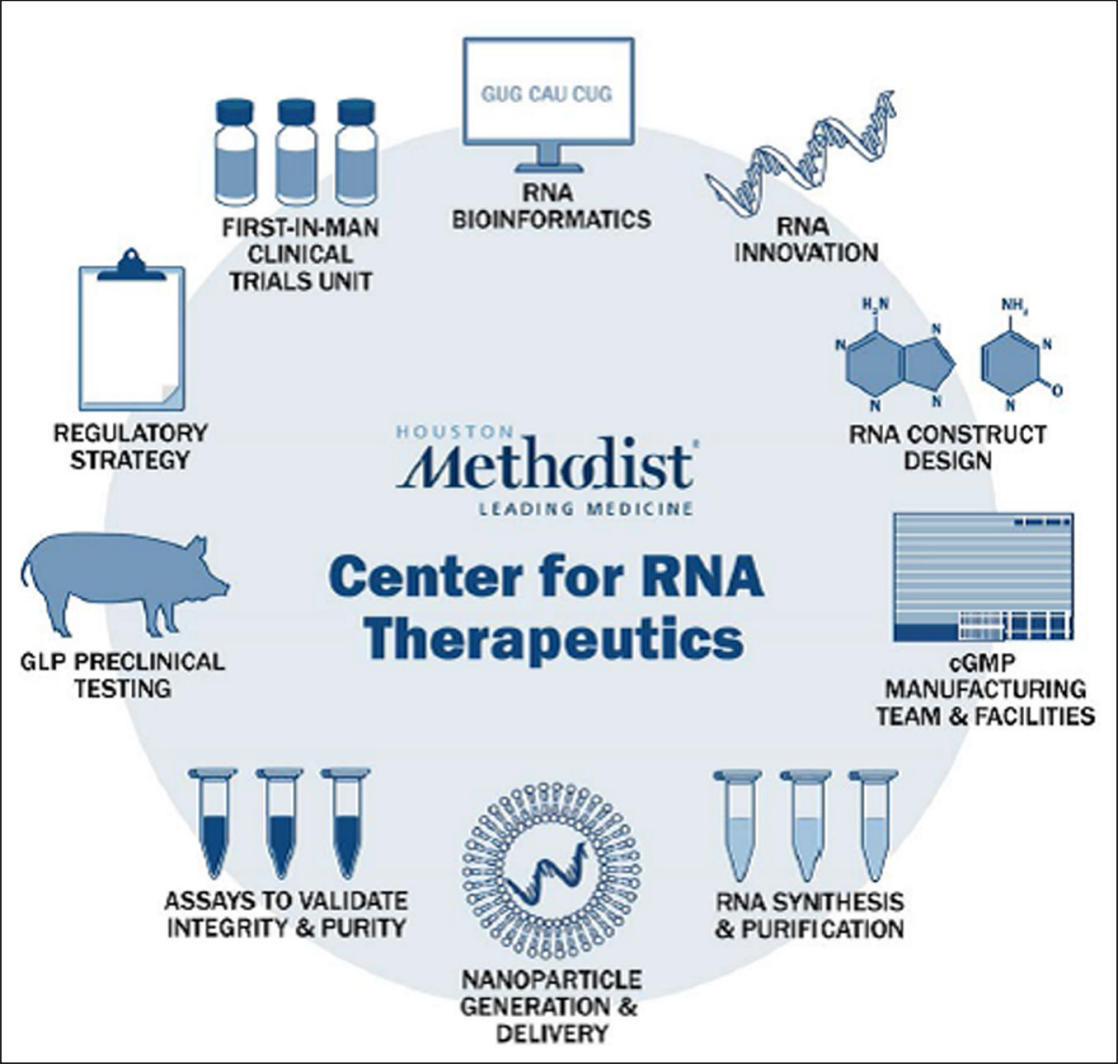
Hospital-based RNA therapeutics. The speed in which therapeutic mRNA can be generated using standard laboratory equipment in facilities with a small footprint facilitates hospital-based mRNA therapeutics. The Houston Methodist Center for RNA Therapeutics (CRT) innovates, designs, and synthesizes research or current Good Manufacturing Practice RNA constructs for internal use as well as for external clients such as biotech startups and academic groups. The center has the technology and know-how to encapsulate the mRNA and provide GLP validation with in vitro assays and preclinical small and large animal studies. In addition, it has a first-in-man clinical trials unit and a large hospital system that routinely performs phase 2 and 3 clinical trials (www.houstonmethodist.org/rna-therapeutics/).

The technology of mRNA therapeutics lends itself to personalized medicine. The code for a therapeutic protein can be quickly written using this biological software and the mRNA rapidly generated by in vitro synthesis using standard laboratory equipment in facilities with a small footprint. In our academic hospital, we have built an assembly line for mRNA therapeutics that is also available for small companies and external academic groups—with a team of scientists who can innovate new approaches; design novel constructs; and synthesize, purify, lyophilize, and encapsulate mRNA constructs into LNPs. We have a small current Good Manufacturing Practice facility to generate therapeutic mRNA for preclinical studies and first-in-man clinical trials. Our comparative medicine program is run by veterinarians with experience in Good Laboratory Practices studies for investigational new drug applications and in working with the FDA. We have a first-in-man clinical trial unit and a large hospital system that supports phase 2 and 3 clinical trials (Figure 2).

Academic hospitals are poised to play an important role in the advancement of mRNA therapeutics as well as their generation and application. Personalized therapies such as cancer vaccines are now truly possible with mRNA therapeutics and are best generated and delivered at an academic center where the patient is receiving care. In this case, biopsy or surgical removal of the tumor would be followed by sequencing to identify tumor antigens. This knowledge permits the generation of mRNA constructs encoding these tumor antigens for administration to the patient as a vaccine. In addition to providing personalized RNA drugs, regional centers of RNA therapeutics can participate in the global response to a pandemic as a rapid reaction force to a local outbreak. Thus, in addition to providing a diverse array of novel therapies for unmet needs, mRNA therapeutics are poised to change the way drugs are developed and delivered to patients. We stand at the threshold of a new therapeutic frontier filled with the promise of discovery and transformational therapies.

## Key Points

- One can think of messenger ribonucleic acid (mRNA) drugs as biological software that encodes therapeutic proteins.
- Therapeutic mRNA has been made possible by understanding that base modifications (such as replacing uracil with pseudouridine) are necessary to make the mRNA drug less toxic.
- In addition, therapeutic mRNA has been made possible by developments in delivery, and in particular the generation of lipid nanoparticles to encapsulate and protect the mRNA from degradation.
- Hospital-based RNA therapeutics programs will provide personalized RNA therapies in the near future, changing the way drugs are developed and delivered.
